# Visual adaptation reveals an objective electrophysiological measure of high-level individual face discrimination

**DOI:** 10.1038/s41598-017-03348-x

**Published:** 2017-06-12

**Authors:** Talia L. Retter, Bruno Rossion

**Affiliations:** 1 Psychological Sciences Research Institute, Institute of Neuroscience, University of Louvain, Louvain, Belgium; 2 Department of Psychology, Center for Integrative Neuroscience, University of Nevada, Reno, USA; 3 Neurology Unit, Centre Hospitalier Regional Universitaire (CHRU) de Nancy, F-54000 Nancy, France

## Abstract

The ability to individualize faces is a fundamental human brain function. Following visual adaptation to one individual face, the suppressed neural response to this identity becomes discriminable from an unadapted facial identity at a neural population level. Here, we investigate a simple and objective measure of individual face discrimination with electroencephalographic (EEG) frequency tagging following adaptation. In a first condition, (1) two facial identities are presented in alternation at a rate of six images per second (6 Hz; 3 Hz identity repetition rate) for a 20 s testing sequence, following 10-s adaptation to one of the facial identities; this results in a significant identity discrimination response at 3 Hz in the frequency domain of the EEG over right occipito-temporal channels, replicating our previous findings. Such a 3 Hz response is absent for two novel conditions, in which (2) the faces are inverted and (3) an identity physically equidistant from the two faces is adapted. These results indicate that low-level visual features present in inverted or unspecific facial identities are not sufficient to produce the adaptation effect found for upright facial stimuli, which appears to truly reflect identity-specific perceptual representations in the human brain.

## Introduction

We are able to differentiate people from visual images of their faces accurately and rapidly (i.e., at a single glance), a necessary process for efficient social interactions in the human species. Despite the ease at which we individualize faces, this process is complex, relying on the extraction from basic visual information of a unique representation that can be used to recognize this face across changes in size, illumination, expression, etc. A better understanding of this process at a neural level would contribute to increasing our understanding of visual discrimination in general.

In order to measure an individual face discrimination response in humans, non-invasive recording techniques such as electroencephalography (EEG) or functional magnetic resonance imaging (fMRI) may be used. However, there is no reason to expect differences in neural responses between individual faces at the level of resolution afforded by EEG or fMRI (i.e., scalp electrodes or voxels). This limitation may explain why studies relying on decoding patterns of neural activity across electrodes or voxels with such techniques generally leads to weak (i.e., barely above chance level) and inconsistent results across studies, even for simple image comparisons without size-invariance (see the extensive discussion in ref. 1; see also ref. 2). In an attempt to transcend these limitations, the response to different facial identities may be differentially amplified, e.g., through visual adaptation to one of the individual faces (e.g., fMRI: refs 3–6; EEG: refs 7–10). With standard stimulation modes and recording, this visual adaptation approach is not without its own limitations, however, in particular when it may produce opposite effects on brain signals, i.e., repetition suppression or repetition enhancement in fMRI (see refs 11 and 12 for review) or changes in multiple event-related potential components with opposing polarity effects in EEG (e.g., N170 amplitude decrease, e.g., refs 8 and 9; N250r increase, e.g., refs 7 and 10).

**Figure 1:**
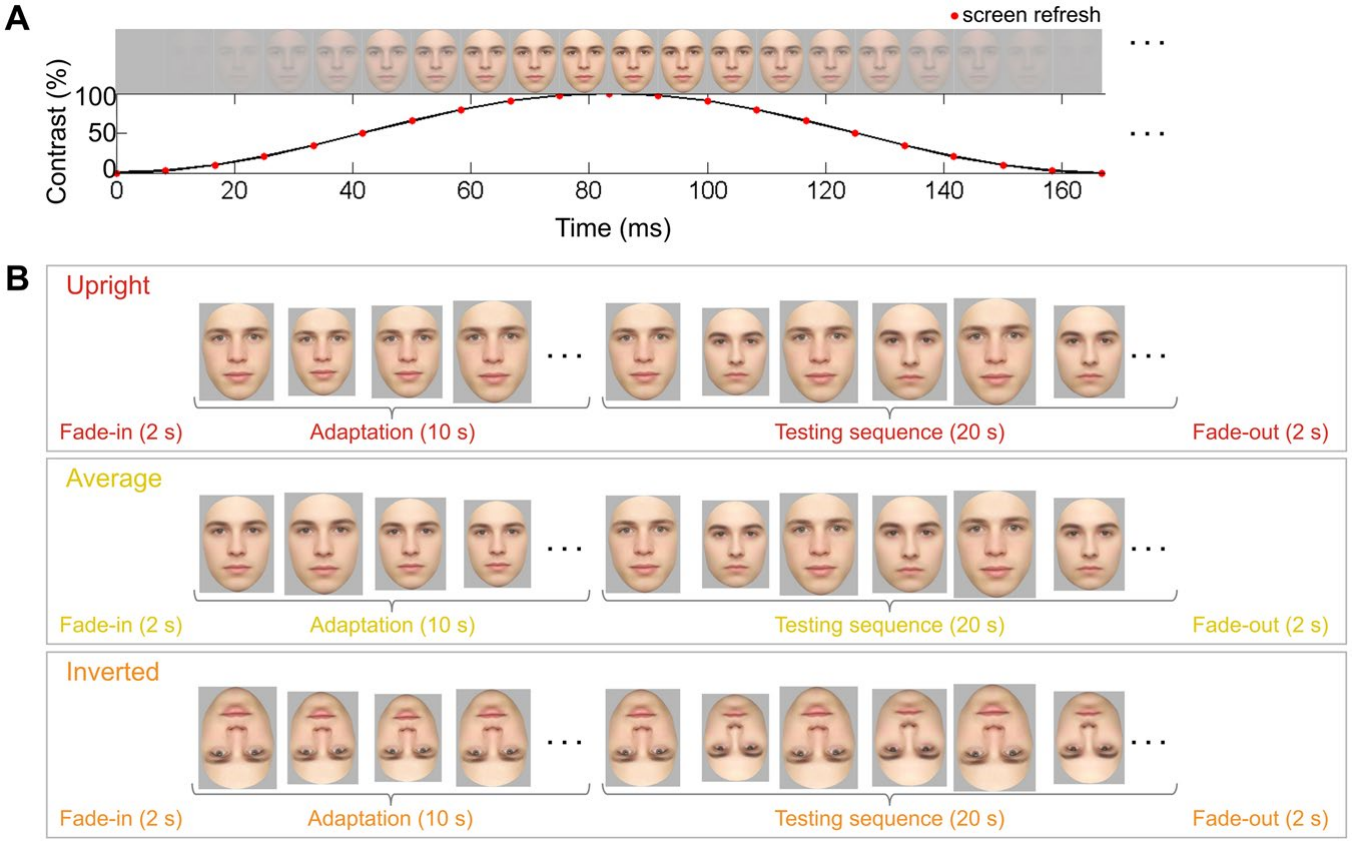
(**A**) Stimuli were presented through a gradual increase and decrease of contrast at each screen refresh frame, following a sinusoidal modulation of image contrast. Given a 120 Hz monitor (i.e., a screen refresh every 8.333 ms) and a 6 Hz stimulus presentation rate (i.e., 166.7 ms per stimulus cycle), each image was presented over 20 contrast steps (166.7 ms/8.333 ms), as illustrated. (**B**) The sequence structure of the three experimental conditions. In all conditions, there was a 10 s adaptation baseline: in the *upright* condition (top row), the adapting identity was one of the identities from the 20 s testing sequence; in the *average* condition (middle row), the adapting identity was not one of the identities from the testing sequence, but the average face; finally, in the *inverted* condition, the adapting identity was one of the identities from the testing sequence but all the stimuli were presented in an inverted orientation. Stimuli were always presented at 6 Hz as depicted in panel A; when a pair of facial identities (a face and its anti-face) were alternated during the testing sequence portion, each identity was thus repeated at a rate of 3 Hz (i.e., 6 Hz/2, corresponding to every 333 ms). Finally, note that at each image presentation cycle, the stimulus size varied from 90–110% of the original image size.

To circumvent such existent issues, we recently introduced a simple adaptation paradigm to objectively define and quantify individual face discrimination responses in EEG^13^ based on fast periodic visual stimulation (FPVS; ref. 14). Such a paradigm, initially developed to study directional selectivity (another process coded for beneath the population level; refs 15 and 16), has the advantage of using periodic stimulation in order to record precise frequency-tagged responses in the EEG (for review: refs 17 and 18). Here, a pair of two different individual faces is shown in alternation at a consistent rate of six images per second (6 Hz, i.e., 167 ms between images; Fig. 1A), producing a response in the frequency-domain EEG spectrum exactly at 6 Hz, reflecting a combination of low- and high-level facial image processing. Critically, following an adaptation period in which one of the individual faces of the pair is presented repeatedly, a response now appears in the EEG exactly at 3 Hz, i.e., the 333-ms rate at which each individual face is shown: this 3 Hz response represents the asymmetry produced between responses to the adapted and unadapted individual faces. The 3 Hz response is consequently a *differential* response, which is not present when comparing two unadapted facial identities^13^. This technique thus provides a means to objectively (i.e., at the frequency determined by the experimenter) identify and quantify (i.e., as the EEG amplitude at 3 Hz corrected for noise level) implicit neural adaptation to a single individual face, as a comprehensive response in the frequency domain at 3 Hz.

In the present experiment, we aim to provide compelling evidence that the adaptation effect at 3 Hz reflects identity-specific adaptation at a high-level representation. To this end, we add two novel conditions to the paradigm (Fig. 1B): first, an *inverted* face condition, in which all the facial identities are presented upside-down. This manipulation preserves low-level visual information but greatly impairs individual face discrimination (e.g., refs 19–21; for reviews refs 22 and 23). In this case, we expect that the limited diagnostic information available from inverted faces is not enough to produce an identity-specific discrimination response after adaptation, or at least a significantly weaker response than for upright faces. We also test an *average* condition, in which a facial identity built to be physically equidistant to the face pairs, and not in the alternating testing pair, is adapted: if adaptation is general, i.e., if adapting to one individual face increases sensitivity to variations across any different individual faces (e.g., *sharpening* the neural representation at a category-level^24^), rather than a specific adaptation to the particular individual face adapted, then an asymmetry in the EEG response may also be observed. Finally, we repeat the original adapted *upright* condition, in which one of the individual faces from the testing pair is adapted in the upright orientation, in order to replicate our previous findings and to quantify the amplitude of the individual face adaptation effect again in this new sample of participants (none of whom participated in the previous study^13^).

## Results

### Asymmetrical identity-specific responses (3 Hz)

#### Frequency-domain response identification

**Figure 2:**
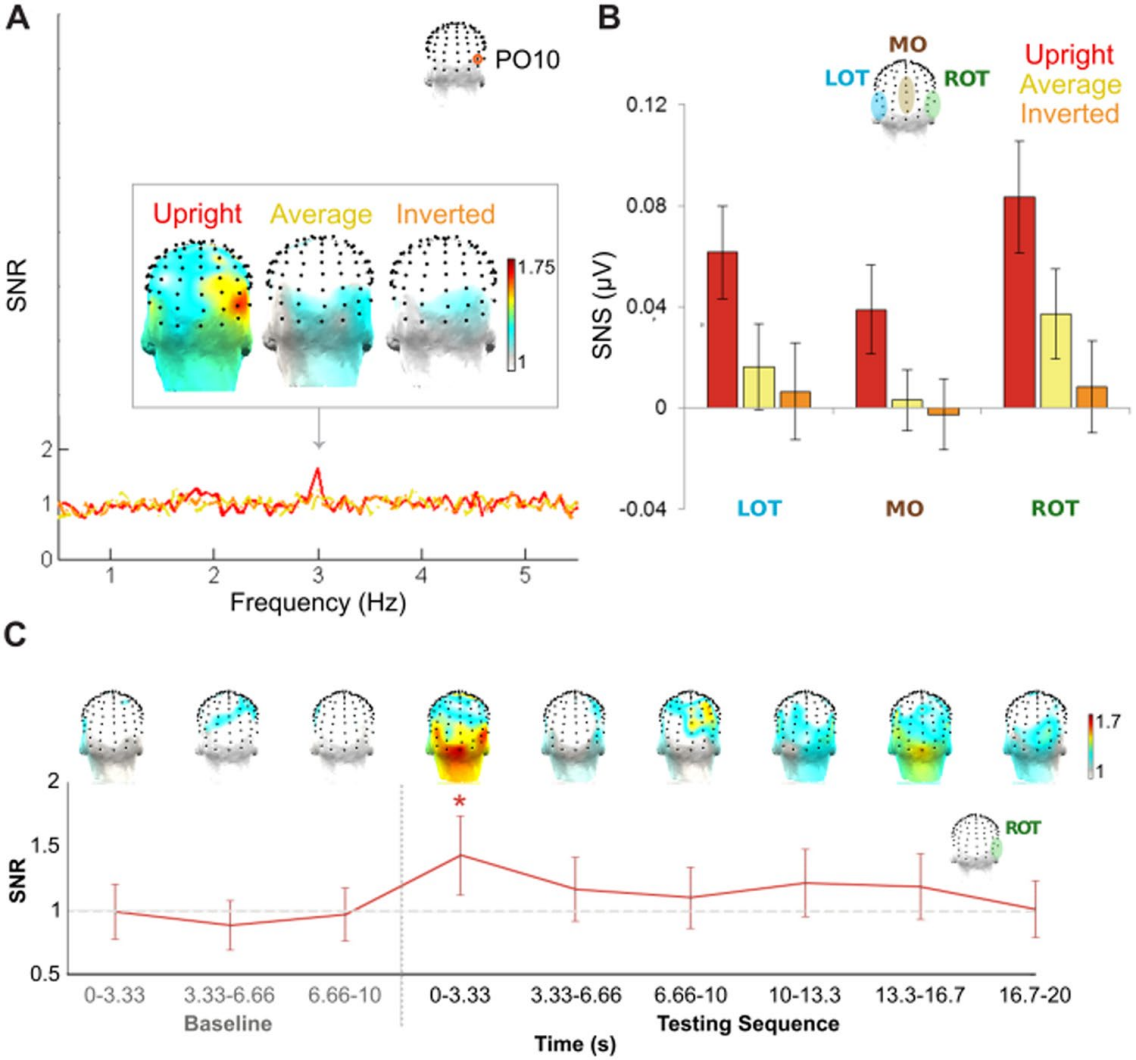
Asymmetrical responses to facial identities at the group level in the EEG frequency domain at 3 Hz following adaptation. (**A**) The signal-to-noise (SNR) spectrum around 3 Hz for channel PO10, one of the channels in the right occipito-temporal (ROT) region-of-interest (ROI), for each of the three experimental conditions; here, a distinct response is observed exactly at 3 Hz only following *upright* adaptation to one of the facial identities of the testing sequence. The scalp topographies of the responses at 3 Hz, shown above, depict the largest activation across the back of the head again for the *upright* condition, with an apparent maximum over the ROT cortex. (**B**) Quantification of the 3 Hz response, in terms of baseline-subtracted amplitude (SNS) over each of the ROIs (LOT: left occipito-temporal; MO: medial-occipital), reveals the largest response for the *upright* condition, occurring maximally over the ROT ROI. (**C**) The temporal evolution of the 3 Hz response present in the *upright* condition: the adaptation effect SNR is greatest over the ROT ROI in the first time segment (3.33 s, chosen arbitrarily) immediately following adaptation (p < 0.01), persisting non-significantly above noise-level (i.e., 1, indicated by a light gray dashed line) throughout the rest of the testing sequence.

The 3 Hz response, indicating an asymmetry (i.e., differentiation) in the response to the two individual faces of a stimulus pair produced by adaptation, was significant for the 128 channel-averaged response only for the *upright* condition, Z = 2.39, p < 0.01; *average*, Z = 0.62, p > 0.05; *inverted*, Z = −0.47, p > 0.05. It appeared most distinctly in the frequency domain for the *upright* condition at right occipito-temporal channel PO10, for which the SNR was 1.67, compared with 1.26 (*average*) and 1.14 (*inverted*) (Fig. 2A). The 3 Hz response was also evaluated at right occipito-temporal (ROT), left occipito-temporal (LOT), and medial-occipital (MO) regions of interest (ROIs; see Methods and Fig. 2B). Over the ROT ROI, at 3 Hz, this response was again only significant for the *upright* condition, Z = 2.54, p < 0.01; *average*, Z = −0.3, p > 0.05; *inverted*, Z = −0.25, p > 0.05: the response at the next specific harmonic, i.e., 9 Hz, was not significant in any condition (all p’s > 0.01).

### Frequency-domain response quantification

Quantification of the individual face-specific response amplitude at 3 Hz showed the response to be largest for the *upright* condition, here being maximal over the *ROT* (0.08 µV), then the *LOT* (0.06 µV), and last the *MO* (0.04 µV) ROI (Fig. 2B). Statistical analysis of the quantified 3 Hz response revealed that there was only a significant main effect of *Condition*, F_(2,20)_ = 5.10, ${\eta }_{p}^{2}$ = 0.34, p = 0.016; however, neither the main effect of *Region*, F_(2,20)_ = 3.00, ${\eta }_{p}^{2}$ = 0.23, p = 0.073, nor the interaction between these factors reached significance, F_(4,18)_ = 0.44, ${\eta }_{p}^{2}$ = 0.089, p = 0.78. Post hoc pairwise comparisons of marginal means with Bonferroni-corrected alpha values for the factor *Condition* showed that the *upright* condition only produced a significantly higher response (0.061 ± 0.014 µV) than either the *average* (0.019 ± 0.012 µV), p = 0.040, or *inverted* condition (0.004 ± 0.013 µV), p = 0.012, as predicted.

### Temporal evolution of the adaptation effect

To examine the temporal evolution of the significant identity-specific discrimination response present in the *upright* condition after adaptation, the baseline and testing sequence were analyzed in successive 3.33 s segments. This segment duration was chosen arbitrarily, within a reasonable range of about 2–5 s given restrictions by the length of the baseline and testing sequences (suggesting a duration evenly divisible in 10 s), as well as the frequency sampling-resolution (the inverse of the segment duration, e.g., 3.33 s leads to a resolution of 0.30 Hz). The results show that the adaptation effect is considerably strongest in the first 3.33 s immediately following adaptation (Z > 2.32, p < 0.01), and persists only slightly, and non-significantly (p’s > 0.01), throughout the rest of the testing sequence (Fig. 2C).

### Temporal Signature of the Adaptation Effect

**Figure 3:**
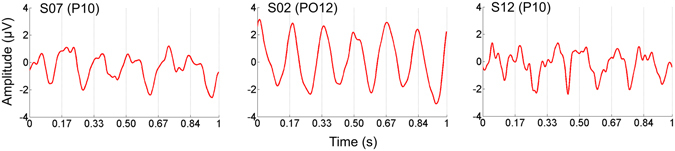
Data from a right occipito-temporal ROI electrode (labeled in parentheses) for three individual participants are shown in the time domain to provide examples of temporal, as well as amplitude, variations in the identity-discrimination response. 0 s represents the onset time of the adapted face sinusoidal cycle onset: adapted faces repeat at 0.33 s and 0.67 s, with unadapted faces presented in between, i.e., at 0.17 s, 0.50 s, and 0.84 s. A somewhat sinusoidal response repeating six times within a second reflects the response to face stimulus presentation at 6 Hz. Asymmetries in the response at 3 Hz, i.e., differences in every other response in magnitude and/or latency, are evidence of differences in the responses to the adapted and unadapted identities.

Beyond investigating the amplitude and temporal evolution of the asymmetry response at 3 Hz in the frequency domain, the data may also be examined in the time domain in order to perceive whether there are contributions from latency differences between the adapted and unadapted faces to the 3 Hz identity-specific discrimination response found for the *upright* adapted condition. Although the response to each individual facial stimulus overlaps with the preceding and subsequent response, producing somewhat sinusoidally-shaped responses, asymmetries are evident in the form of different magnitudes and/or latencies between every other response cycle: these contributions appear variable across participants (Fig. 3).

### Symmetrical stimulus-presentation responses (6 Hz)

**Figure 4:**
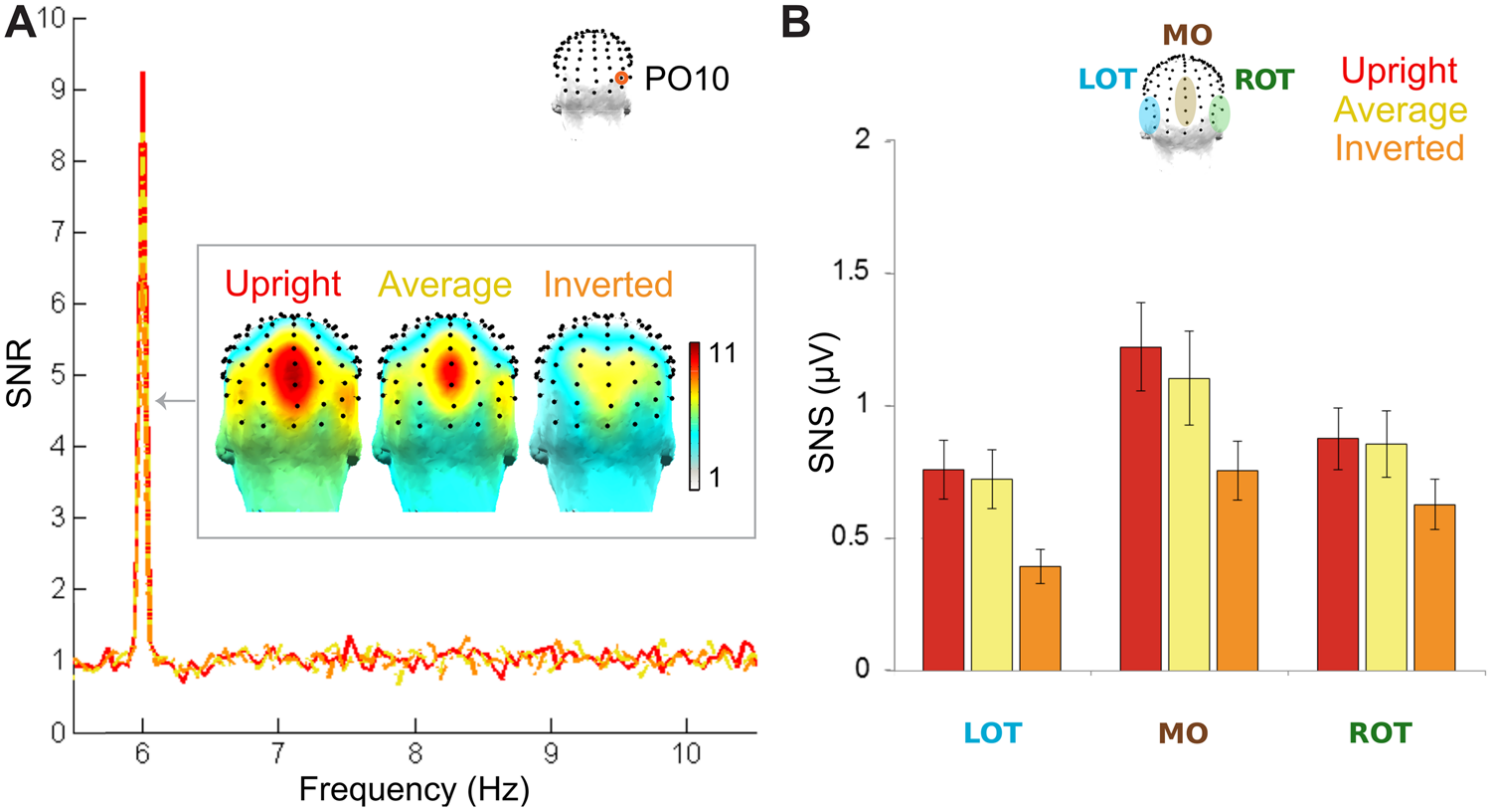
Symmetrical responses to the 6 Hz stimulus-presentation rate at the group level in the EEG frequency domain. (**A**) Frequency-domain signal-to-noise (SNR) spectra of a single channel (PO10, one of the channels in the right occipito-temporal (ROT) ROI) are shown for the three conditions, continued from Fig. 2A, along with the topographical scalp maps of the 6 Hz response, summed across its harmonics. The response at 6 Hz is distinct in the spectrum for all conditions. It consistently appears maximally over the medial occipital (MO) region, despite some topographical differences across conditions. (**B**) Quantification of the mean response in terms of baseline-subtracted amplitude (SNS) over each of the ROIs at 6 Hz and its five additional significant harmonics, up to 36 Hz. The response indeed occurs maximally over the MO region in all conditions, although the *inverted* condition shows overall lower responses and a ROT amplitude similar in amplitude to that of its MO ROI.

Responses at the stimulus-presentation frequency, i.e., 6 Hz, were found clearly in all conditions (Fig. 4A). These responses were maximal over medial-occipital (MO) regions, indicating a primarily low-level response. Analysis of the average of all 128 channels revealed highly significant responses for every condition at 6 Hz (*upright*: Z = 47.2, p < 0.001; *average*: Z = 58.5, p < 0.001; *inverted*: Z = 79.4, p < 0.001). At the maximal MO ROI, the response was significant at six harmonic frequencies (from 6 to 36 Hz) in both the *upright* and *average* conditions, and at five harmonics in the *inverted* condition (all p’s < 0.001): further analyses on the quantified response will reflect the sum of the SNS responses at these six harmonics for all conditions (see Methods; Fig. 4B).

There were significant main effects of both *Condition*, F_(1.5,15)_ = 32.9, ${\eta }_{p}^{2}$ = 0.77, p < 0.001, and *Region*, F_(1.4,14)_ = 13.6, ${\eta }_{p}^{2}$ = 0.58, p < 0.001, with no significant interaction, F_(1.7,7.8)_ = 2.50, ${\eta }_{p}^{2}$ = 0.36, p = 0.079. Post hoc pairwise comparisons of marginal means Bonferroni-corrected for multiple comparisons for the factor *Condition* revealed that both the base responses to stimulus presentation for the *upright* (1.39 ± 0.17 µV) and for the *average* condition (1.31 ± 0.18 µV) were significantly larger than that for the *inverted* condition (0.87 ± 0.12 µV), both p’s < 0.001; the response for the *upright* condition was not significantly different in magnitude from that for the *average* condition (p = 0.14). Equivalent comparisons of marginal means for the factor *Region* showed that the stimulus-presentation responses were larger over the MO ROI (1.50 ± 0.21 µV) than over the LOT ROI (0.92 ± 0.14 µV), p < 0.001, but differences were not significant in response amplitude between the MO and ROT ROIs (1.15 ± 0.16 µV), p = 0.17, or between the ROT and LOT ROIs, p = 0.072.

## Discussion

Adaptation to an upright individual face image (*upright* condition) for 10 s subsequently produced a differentiable response to that face and its anti-face of the same sex in a 20 s testing sequence, measured from the frequency domain of the EEG exactly at the identity-repetition rate of 3 Hz. These results replicate that of a previous study by the present authors using this FPVS-EEG adaptation paradigm, in which adaptation produced a 3 Hz identity-specific response which was absent for unadapted facial identities in 16 observers^13^. Importantly, the identity-specific response found here was also not present in two novel conditions, in which 1) face stimuli were presented in an inverted orientation (*inverted*) or 2) following adaptation to an average face (*average*).

In the *inverted* condition, the 3 Hz identity-specific asymmetry response was not significant across all channels or at the ROT ROI; across all three ROIs (see Methods; Fig. 1B) its amplitude appeared trivial, falling below 0.01 µV. The lack of adaptation-induced asymmetry to inverted faces provides evidence that the 3 Hz response here is high-level and identity-specific: even while low-level visual information is preserved in inverted stimuli, this suboptimal orientation for engaging the expert facial identity-processing system (e.g., refs 20–23) is not enough here to produce a high-level representation of identity. This is line with identity-specific responses being found mainly for upright, and not inverted, faces in FPVS-EEG (e.g., ref. 24), as well as with dramatically reduced identity after-effects across participants in behavior^25^. Interestingly, it may be noted that inversion has had mixed outcomes compared to upright faces in identity after-effect experiments behaviorally (e.g., ref. 25 vs. ref. 26; see ref. 27 for a review). One possible account for this discrepancy is that the present paradigm measures face identity discrimination with tight temporal constraints (as will be addressed later in this discussion). Hence, while behavioral after-effect responses likely reflect the outcome of many general processes which are shared between upright and inverted faces (despite the use of about 200 ms of test stimulus duration, response time is typically unconstrained), our paradigm may be better suited for isolating (fast) perceptual processes related to individualization of faces.

In the *average* condition, in which adaptation was induced for the average face, rather than for one of the faces in the testing pair, the 3 Hz identity-discrimination response was also not significant across all channels or at the ROT ROI. Here, however, the amplitude of the response was significantly but only moderately lower, i.e., about 40%, than in the *upright* adapted condition over the maximal ROT ROI. Thus, although the *average* 3 Hz response was not significant, there may be some weak evidence of an average face increasing sensitivity to different facial exemplars in a general fashion (see ref. 26 for similar evidence at a behavioral level). This would support adaptation to any face “sharpening” the representation of different individual facial identities^28^, albeit at a category- rather than individual exemplar-level. Importantly, the adapting face used here was the average of the testing face pair, such that it represented the equidistant point of physical similarity to either face, according to the dimensions of shape and texture along which it was created through stimulus morphing (e.g., ref. 29). However, it is still possible that the average face carried more perceptual similarity to one of the faces in the testing pair than the other (e.g., typical vs. atypical faces^30^), which could also have induced some bias in the direction of adaptation for one of the facial identities. In either case, the lack of significant response for this *average* adaptation again serves to provide evidence that the response for the *upright* condition is truly supported by high-level identity-specific adaptation effects.

In contrast to the striking differences across conditions in the 3 Hz response, responses to general face stimulus presentation at 6 Hz and its harmonics were found in all three conditions. The *average* condition 6 Hz response amplitude was not significantly different from that of the *upright* condition; however, in the *inverted* condition, the response was significantly reduced relative to both the *upright* and *average* conditions. While we must remain speculative at this stage, one possible explanation for this decrease in the *inverted* 6 Hz response following adaptation is a lack of enhancement of the representation of lower-level differences for purposes of identity for inverted faces at this presentation rate, thus allowing adaptation to be generalized across the two stimuli. Another possibility is that the 6 Hz response, which largely reflects the general response to visual stimulation (i.e., the contrast between a face and a background), is larger for upright than inverted stimuli due to the larger contrast in the upper visual field (e.g., projecting to the lower calcarine sulcus) for upright faces.

In addition to introducing two novel conditions, the present experiment served to replicate previous findings of identity-discrimination responses at 3 Hz following adaptation to upright faces^13^. This replication was produced in an entirely new sample of participants: the 3 Hz response following adaptation in the *upright* adapted condition here was significantly present at the group level over the 128-channel average as well as over the right occipito-temporal (ROT) ROI. Its amplitude peaked at ROT channel PO10. Furthermore, the amplitude of the 3 Hz response was found to be similar to that of the first study^13^: considering the two studies together, the ROT ROI showed the maximal response, and the predicted population amplitude over this ROI is predicted to be in the range of about 0.09 to 0.1 µV. Note that while this may seem to be a modest response amplitude, it corresponds to a SNR of 1.67, i.e., a 67% increase of signal relative to the surrounding noise level (Fig. 2A).

The distribution of the 3 Hz response on the scalp here also appears to be similar across the current and previous study. Here, the response was twice as large over the ROT ROI as the response over the MO ROI, and 0.33 times larger than the response over the LOT ROI. In the previous study (see Fig. 4B of ref. 13), the response at the ROT ROI was 2.3 times larger than at the MO ROI, and 0.55 times larger than the response over the LOT ROI. It may be observed that the ROT vs. MO ROI comparison is more consistent across the two studies than the ROT vs. LOT ROI comparison, perhaps indicating greater consistency in the contributions of right lateralized face-specialized processes relative to lower-level visual facial identity responses across participants than in higher-level hemispheric lateralization. The maximal ROT response localization found at the group level here reflects a human right hemispheric dominance for face perception as typically observed at the group-level with low-spatial resolution scalp EEG^31, 32^, and more precisely with intracerebral EEG^33^, positron emission tomography (PET^34^) and fMRI^35–37^. Most importantly, lesion studies have consistently shown that a right but not a left ventral occipito-temporal lesion may be both necessary and sufficient to cause prosopagnosia, i.e., a severe and sometimes specific impairment at individual face recognition^37–39^; ref. 1 for review.

For the response to face stimulus presentation at 6 Hz and its harmonics (up to 36 Hz), similarities between the current and previous study^13^ were also evident in terms of overall response amplitudes and patterns across the scalp. The response amplitude at the maximal ROI, the MO ROI, was very similar across the two studies (about 1.7 to 1.9 µV). The distribution of the response across the scalp was also consistently similar across these studies, with the MO ROI response 0.39 times larger than the ROT ROI in the previous study vs. 0.34 times larger here, but the ROT ROI response being 0.31 times larger than the LOT ROI response in the previous study and only 0.08 times larger here. Again, these results indicate a greater consistency across subjects in the relative occipito-temporal vs. medial occipital responses, indicating a consistent relationship between these areas independent of which region is dominant for the type of stimulation presented.

The 3 Hz asymmetry in response to an adapted and unadapted identity found in the *upright* condition here was further explored in terms of its temporal dynamics. Not present merely in terms of quantified amplitude, it is also apparent in shifts of latency at the level of individual participants (Fig. 3; compare to Fig. 6 of ref. 13); although, here, given overlapping responses in time due to the fast image presentation rate of 6 Hz (see the discussion in ref. 40), latency shifts cannot be attributed to either one of the stimuli independently. However, any latency differences to the two facial identities may give some slight support to a *facilitation* model of the neural mechanisms subtending this adaptation effect, wherein adaptation decreases the relative response latency to the adapted stimulus^28^. The temporal duration of this response here, as in the first experiment, is shown to be strongest in the first few seconds following adaptation; after at most 6 s, it persists non-significantly until near the end of the 20 s testing sequence. Such a rapid decay of an adaptation effect is in line with behavioral studies, which show an inverse relationship between the time of adaptation the duration of the adaptation effect, which declines exponentially with test duration (see ref. 41).

Finally, we turn the discussion to the fast periodic visual stimulation (FPVS) technique used here, which provided several important advantages. Among these are (1) *objectivity*, in that a response is localized at a precise location in the frequency-domain spectrum, pre-defined by the image presentation frequency; (2) *sensitivity* provided by the high signal-to-noise ratio of the frequency-domain analysis; (3) *isolation of functionally specific responses*, e.g., responses to face presentation vs. facial identity, due to frequency-tagging within the same testing sequences; (4) *quantification* of a comprehensive response in the frequency domain, independent of individual response components (see ref. 13); (5) *implicit* response measurement, as participants perform an orthogonal task (see refs 14 and 18 for reviews). Additionally, the present paradigm takes advantage of separating responses of interest from low-level variations across stimuli: image properties may be varied throughout image presentation (e.g., here luminance contrast is varied at each screen refresh according to a sinusoidally modulated increase/decrease) and at each stimulus presentation cycle (e.g., here size is varied randomly in five steps from 90–100% of the original image size), such that these low-level properties do not appear consistently at the stimulus presentation rate, i.e., are not represented in the frequency-locked response to stimulus presentation.

One limitation of the FPVS-EEG design, at least with relatively short SOAs as presented here (i.e., about 167 ms between face stimuli, and 333 ms between identity repetitions), is a decreased temporal resolution compared to traditional ERP studies. The response here in the time domain appears mostly sinusoidal, or at least responses to different stimuli are clearly overlapping, such that the responses to each individual identity cannot be dissociated (Fig. 3). (Given that the response at 3 Hz represents the response to the repetition of both of the facial identities presented in this paradigm, neither can a unique frequency can be used to isolate the response to either stimulus). Therefore, it is difficult to attribute amplitude or latency differences to one of the stimuli, e.g., whether an asymmetry at 3 Hz reflects a decrease in the response to the adapted face or an increase in the response to the unadapted face, or both; however, some timing information can still be extracted from the phase of the response (see ref. 13).

The relatively short SOAs between stimuli at a 6 Hz presentation rate has raised questions of whether the neural responses to each stimulus are too temporally constrained (e.g., ref. 42). However, in previous studies, 5.88 Hz has been shown to produce the maximal amplitude difference between presentations of different identities or the repetition of the same identity at every image presentation cycle, indicating not only that it provides enough time for identity discrimination, but that it is optimal for achieving adaptation effects in EEG^43^ and in fMRI^4^. One reason that identity may be perceived at this rate is that the neural response time is not limited to the image presentation time, but to the restriction put on processing one stimulus by the appearance of the subsequent stimulus^40^. According to this view, a 167 ms SOA does not restrict the processing of a stimulus to 167 ms; rather, the processing time is interrupted only when competitive levels of processing overlap, with later processes of a previously presented face perhaps being distinct from the early processes in the response beginning to the next face presented. Note that here, identity-specific stimuli that may produce fully identical responses only repeat every 333 ms. There are several other reasons to use an image presentation rate of 6 Hz: it matches the minimum amount of time needed to optimally identify faces^44^, limiting variable extraction of social information across participants and so reducing irrelevant inter-individual variability^45, 46^; additionally, it allows for only a single gaze fixation at each image presentation cycle, therefore eliminating gaze exploration of each image (a potential reason for observing much larger and consistent inversion effects than behavioral studies of adaptation^27^) and reducing eye movement artifacts.

In conclusion, a replicable, high-level, identity-specific adaptation response to upright facial stimuli can be found after less than five minutes of EEG recording from each participant. Importantly, this effect is not present without adaptation^13^, nor for inverted faces, nor for adapting to an average face stimulus. Thus, this technique may be considered as a tool to be applied in future studies to investigate the perceptual underpinnings of identity-specific discrimination by careful manipulation of adapting and testing facial stimuli, e.g., to explore normative coding of faces, familiarity, attractiveness, or the relation of different facial expressions. Given the short experimental duration and the lack of explicit response required with this FPVS-EEG technique, this paradigm may also be extended to use with infants, children and clinical populations (e.g., respectively: refs 47–49).

## Methods

### Participants

Twenty-two healthy participants (age range 19–25 years; nine female) recruited from a university campus took part in this experiment, a completely independent sample from^13^. They reported normal or corrected-to-normal vision, were all right handed according to an adapted Edinburgh Handedness Inventory measurement (lowest right-handedness score: 45/50; ref. 50), and their Benton Face Recognition Test (BFRT; ref. 51) scores were in the normal range, from 40/54 to 49/54, i.e. 74 to 91% in accuracy; M = 84%, SE = 1.04%. Participants gave signed, informed consent before the start of the experiment, which was approved by the Biomedical Ethical Committee of the University of Louvain and run in accordance with the guidelines and regulations of this committee and the Declaration of Helsinki. They received monetary compensation for the time of their voluntary participation.

### Stimuli

Eight face/anti-face pairs were used (half female; see Fig. 1 for examples; see also Fig. 1 and the Methods of ref. 13). These stimulus pairs were used in order to standardize a relatively large amount of difference within each set of facial identities (for more details, see ref. 13). They were presented exactly as in the previous study, in color with a base presentation size of approximately 5.0 degrees of vertical visual angle. While in the *upright* and *inverted* conditions no additional stimuli were introduced, two novel stimuli were additionally presented here in the *average* condition: the average female and average male face. These stimuli were created from the average of the 12 female and 12 male facial identities, respectively, used in the creation of the face/anti-face pairs with *JPsychoMorph* software^29^.

### Procedure

Participants were tested during the day in a quiet, light-attenuated room of a university building. After filling out a few preliminary questionnaires and preparation of the EEG recording (about 45 minutes in total) they were seated at a viewing distance of 80 cm from an LCD monitor with a resolution of 1920 by 1080 pixels and a screen refresh rate of 120 Hz, and with a keyboard to give responses. Participants were instructed to attend to the presented stimuli while fixating on a superimposed, centrally presented fixation cross: to encourage constant attention, participants were asked to press on the space bar each time that the fixation cross briefly changed color (200 ms), from roughly equiluminant blue to pink, which occurred randomly four times within each trial, i.e., about every 8.5 s. There were no differences across conditions in response accuracy (*upright*: M = 94%, SD = 6.7%; *average*: 96%, SD = 4.6%; *inverted*: M = 95%, SD = 7.2%), F_(2,20)_ = 0.65, ${\eta }_{p}^{2}$ = 0.06, p = 0.53, or response time (*upright*: M = 442 ms, SD = 49.2 ms; *average*: 446 ms, SD = 50.6 ms; *inverted*: M = 455, SD = 55.0 ms), F_(2,20)_ = 2.99, ${\eta }_{p}^{2}$ = 0.23, p = 0.073, suggesting consistency in attention throughout the experiment.

There were 24 trials of 34 s each, for a total testing time of about 20 minutes, with short breaks given between trials. In every trial, stimuli were presented every 166.7 ms, i.e., at a rate of 6 Hz (i.e., 1/0.1667 s). Each image was presented gradually through sinusoidally increasing and decreasing the luminance contrast at each 8.333 ms screen refresh: thus, in each image presentation cycle, the contrast increased in the following contrast steps: 0, 2.45, 9.55, 20.6, 34.5, 50, 65.5, 79.4, 90.5, 97.6, and 100% (see Fig. 1A). Stimuli were presented with carefully controlled timing through Psychtoolbox 3.0.9 for Windows (see ref. 52), running over MATLAB R2009a (MathWorks, USA). In addition to the low-level features of the stimulus thus varying at every screen refresh rate, to further reduce possible low-level adaptation, the size of each stimulus varied randomly at each presentation cycle among five steps in a range of 90–110% of the original presentation size^53^.

The structure of each trial was the same across conditions (Fig. 1B). Each trial began with designs to reduce expectation and reaction to sudden stimulus-onset: a jittered 2–5 s of a grey background and a 2 s stimulus fade-in, during which the maximum stimulus contrast gradually increased to 100%. There was then a 10 s adaptation baseline, in which the same facial identity was presented repeatedly at 6 Hz, followed by the testing sequence of 20 s of alternation of two facial identities from a face/anti-face stimulus pair (for an identity repetition rate of 3 Hz, i.e., 6 Hz/2, corresponding to 333 ms). Finally, to similarly, gradually end each trial, there was a 2 s stimulus fade-out and 2 s of the grey background.

Three experimental conditions were tested (8 trial repetitions each). In the adapted *upright* condition, one facial identity from the 20 s testing sequence pair was presented over the 10 s adaptation baseline (exactly as in the “adapted” condition of ref. 13). In the adapted *average* condition, an individual face which did not appear in the 20 s testing sequence (i.e., the average face) was used in the adapting baseline, matching the sex of the face pair in the testing sequence. In the adapted *inverted* condition, the sequence structure (including both adapting and testing sequence portions) was the same as in the *upright* condition, except that the faces were presented in an inverted orientation, i.e., at a 180 degree rotation from upright.

The eight face/anti-face pairs were distributed across the three conditions in two trial lists, which were counterbalanced across participants. This was done in order to use the same identity pairs as much as possible, standardizing the amount of perceptual difference between facial identities across trials, while reducing potential identity familiarization effects across trials, particularly for upright image presentation in the *upright* and *average* conditions. In the first trial list, half of the individual face pairs were presented in the *upright* condition (pairs 1, 2, 3 and 4; half male) and the other half in the *average* condition (pairs 5, 6, 7, and 8; half male); the odd-numbered individual face pairs were repeated in the *inverted* condition. In the second trial list, the individual face pairs in the *upright* and *average* conditions were swapped, and the even-numbered individual face pairs were repeated in the *inverted* condition. Each individual face pair was presented two times in each condition, one with the face as the adapting baseline stimulus and the other with the anti-face as the adapting baseline stimulus. The identity used in the adapting baseline always appeared first in the testing sequence. Trial order was randomized fully for each participant separately.

### EEG Acquisition

A BioSemi ActiveTwo system with 128 Ag-AgCl Active-electrodes was used. These electrodes were positioned in the default BioSemi configuration, centered around nine standard 10/20 locations on the primary axes (BioSemi B.V., Amsterdam, Netherlands; for exact electrode location coordinates, see http://www.biosemi.com/headcap.htm). Electrode labels from BioSemi were relabed to match closely with the 10/5 system^54^ (e.g., A1 was relabeled to Cz and B8 to PO10; for all relabeling, see ref. 55, Fig. S2). In addition, four flat-type Active-electrodes were attached above and below each participant’s right eye and lateral to the external canthi in order to record, in respective pairs, the vertical and horizontal electrooculogram (EOG). Electrode offsets, referenced through the common mode sense (CMS) and driven-right leg (DRL) loop, were held below 40 mV. The EEG and EOG were digitized with a sampling rate of 512 Hz.

### Analysis

#### Frequencies-of-Interest

Frequency-locked responses were expected at the stimulus-presentation rate (*F*), i.e., 6 Hz, reflecting a combination of low-level and high-level responses, including face-selective responses^52, 54^. Additional harmonic-frequency responses, i.e., 2 *F*, 3 *F*, etc., were also expected for this base stimulus-presentation response, up to about 6 *F*, i.e., 36 Hz^40^. In this paradigm, adapting to one individual face from a stimulus pair is expected to produce an asymmetry in the responses to the two individual faces of that pair (one adapted and one not), presented in alternation throughout the testing sequence^13, 15, 16^. Therefore, if adaptation were to produce an identity-discrimination response, it would be expected exactly at the individual face-repetition rate of 3 Hz.

#### Regions-of-Interest

Although an analysis on all channels averaged was carried out, three regions-of-interest (ROIs) of five channels each were also determined for more spatially specific analyses of responses, based on both the previous study and the observed scalp topographies here, over the right occipito-temporal (ROT) cortex, homologous left occipito-temporal (LOT) cortex, and the medial occipital (MO) cortex^13^. These ROIs were comprised of the following channels: PO10, P10, PO12, P8, PO8 (ROT); PO9, P9, PO11, P7, PO7 (LOT); and PPOz, POz, POOz, Oz, Oiz (MO). The ROT ROI was defined to capture the expected high-level identity-specific response at 3 Hz, while the MO ROI was designed to reflect the maximal stimulus-presentation response at 6 Hz^13, 52, 55^.

#### Preprocessing

The data of each participant, recorded in a single BioSemi Data Format (BDF) file, was imported into Letswave 5, an open source toolbox (http://nocions.webnode.com/letswave), running over MATLAB R2013b (MathWorks, USA). Since the recording was paused during breaks in between trials (with pause times indicated by an Epoch trigger), resulting changes in drifting electrode offsets were corrected by aligning the offset of each channel immediately after the pause to its prior offset. The data were then filtered with a fourth-order zero-phase Butterworth band-pass filter, with cutoff values of 0.1–120 Hz, and a Fast Fourier Transform (FFT) multi-notch filter, with a width of 0.5 Hz, to remove three harmonics of the 50 Hz electrical noise. Next, the data were segmented by trial, including one second before and after the time of the stimulus fade-in and fade-out, respectively. Channels which were artifact-prone across multiple trials (0 to 5 channels per participant; 1.3% of channels on average) were interpolated linearly with 3 to 5 pooled neighboring channels. Finally, all EEG channels were re-referenced to the common average.

#### Frequency Domain Analysis

The data were re-segmented to an integer number of 3 Hz cycles during the testing sequence, i.e., 59 cyles of 0.3334 s, for a total of 19.67 s, and averaged within each condition for each participant. A FFT was computed on the data from each electrode, transforming the data from the time-domain to a frequency-domain spectrum (frequency resolution of 0.05 Hz; range 0 to 256 Hz) in the form of normalized amplitude (µV).

To determine significance of a response at the frequencies of interest and their harmonics at the group level, Z-scores (Z = (x-baseline)/standard deviation of the baseline) were computed from the grand-averaged amplitude spectra, using a baseline range of about 1 Hz around the frequency bin of interest (x), i.e., the twenty bins closest to the frequency of interest, excluding the immediately adjacent bins^39, 56, 57^. This was done for the amplitude spectra averaged across all 128 channels, to discover significant responses across the whole scalp, as well as for the ROI where the maximal response was predicted (i.e., the MO ROI for the 6 Hz response and harmonics, and the ROT ROI for the 3 Hz response), to ascertain the range of significant harmonic responses across conditions. A response was considered to be significant if Z > 2.32, i.e., p < 0.01.

Quantification of the response was performed for each participant over each ROI by summing the significant harmonics of the adaptation response at 3 Hz and the stimulus-presentation response at 6 Hz separately, after accounting for variations in baseline noise across the frequency spectrum by applying a baseline-subtraction (i.e., a signal-noise subtraction, SNS)^39^. The baseline period was the same as for the Z-score computation, using twenty bins, except that here the maximum and minimum bin were excluded. Additionally, for a display emphasizing the proportion of signal not accounted for by the noise, this baseline was divided from the signal of interest, i.e., the signal-to-noise ratio (SNR) was calculated (e.g., refs 13 and 56). For display at the group level, the SNS and SNR spectra were each averaged across participants.

Statistical comparisons of the three experimental conditions were performed separately for identity-specific (3 Hz) and stimulus-presentation (6 Hz) responses with two-way repeated measures analyses of variance tests (ANOVAs), with factors of *condition* (upright, average, inverted) and *region* (LOT, MO, ROT). A Greenhouse-Geisser correction was applied to the degrees of freedom when Mauchly’s test of sphericity was significant.

#### Temporal Evolution of the Adaptation Effect

In order to investigate the temporal evolution of the significant 3 Hz response over time, the pre-preprocessed (re-referenced) data were segmented arbitrarily into 3.33 s pieces, subsisting of three pieces for the adapting baseline and six pieces for the testing sequence (see Results). These pieces were averaged in time before a FFT was applied (frequency resolution: 0.3 Hz). A SNR baseline-correction was applied: given the lower frequency resolution, the baseline noise, defined as about 1.5 Hz around the signal of interest, constituted four bins after excluding the local maximum and minimum amplitude bins; a Z-score was computed using only the four adjacent frequency bins to the bin of interest.

#### Time-Domain Analysis

Refererenced preprocessed data were more conservatively low-pass filtered above 30 Hz with a fourth-order, zero-phase Butterworth filter, as is common in traditional ERP analyses (e.g., ref. 58). The data were segmented into non-overlapping 1 s epochs within each 20 s testing sequence; this led to 152 epochs per condition, averaged per participant.
